# Supplementary material for: Sustainable Particleboards Based on Brewer’s Spent Grains
Source: Polymers (Basel). 2023 Dec 23;16(1):59. doi: 10.3390/polym16010059 (PMC10780620; doi:10.3390/polym16010059)
Supplement: Supplementary file 1 [file polymers-16-00059-s001.zip › polymers-2782658-supplementary.pdf]

Supplementary material

Text S1. Thermogravimetric analysis

The thermal stability of the BSG was determined using thermogravimetric analysis (TGA) in order to fix the temperature window during pressing. TGA was conducted using a TGA50 Shimadzu (Tokyo, Japan), at 10°C/min under nitrogen atmosphere (flow rate 200 mL/min). The sample weight in all tests was approximately 7-10 mg using platinum crucibles. The initial decomposition temperature (IDT) was identified as the point of onset of weight loss, occurring at 1% (after the moisture loss), while the residual mass percentage was determined at 700°C [1]. Additionally, derivative thermogravimetric analysis (DTGA) was employed to identify temperature peaks associated with various degradation processes.

Figure S1 show the TG and DTG curves under N_2 atmosphere of BSG. The initial weight loss observed around 100°C is attributed to the evaporation of water from the sample. IDT and residual mass values were 193°C and 21 %, respectively. Early degradation of residual sugars and proteins can occur starting from 200°C [2]. Furthermore, the two peaks at 293°C and 351°C in the DTG curve correspond to the degradation of hemicellulose and cellulose, respectively [3]. These events coincide with the extended decomposition of lignin, spanning from 250 to 500°C [2]. The high peak at 293 °C could also indicate a higher content of hemicellulose compared to cellulose [3].

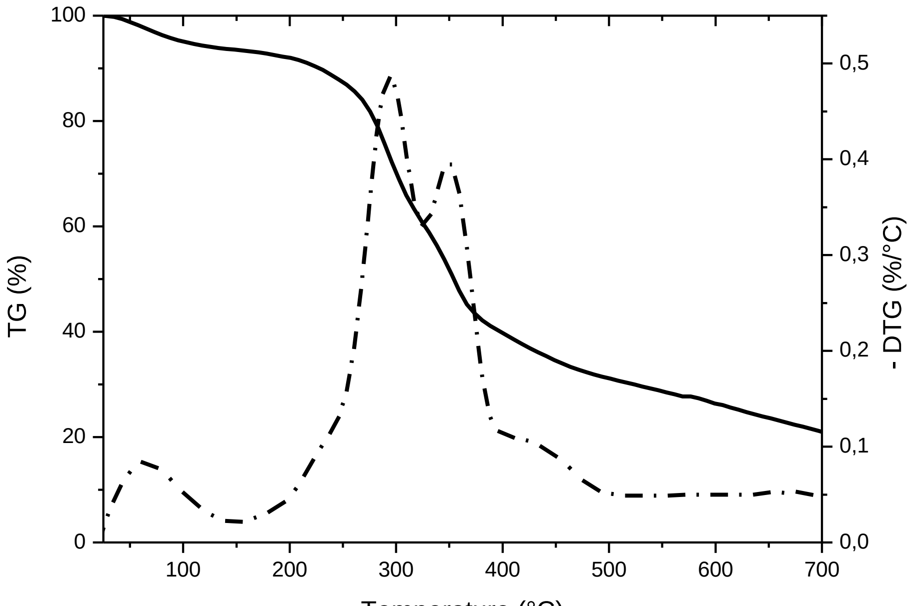

Figure S1: (solid line) TG and (dash-dot line) DTG curves of BSG

References

1. Stefani, P.M.; Cyras, V.P.; Tejeira Barchi, A.; Vazquez, A. Mechanical Properties

and Thermal Stability of Rice Husk Ash Filled Epoxy Foams. *J. Appl. Polym. Sci.* **2006**, *99*, 2957–2965.

2. Mishra, P.K.; Gregor, T.; Wimmer, R. Utilising Brewer's Spent Grain as a Source of Cellulose Nanofibres Following Separation of Protein-Based Biomass. *BioResources* **2017**, *12*, 107–116, doi:10.15376/biores.12.1.107-116.

3. Borel, L.D.M.S.; Lira, T.S.; Ribeiro, J.A.; Ataíde, C.H.; Barrozo, M.A.S. Pyrolysis of Brewer's Spent Grain: Kinetic Study and Products Identification. *Ind. Crops Prod.* **2018**, *121*, 388–395, doi:10.1016/j.indcrop.2018.05.051.
